# Oral leukoplakia: still an enigmatic disorder

**DOI:** 10.4317/medoral.27214

**Published:** 2025-03-23

**Authors:** José Manuel Aguirre-Urizar

**Affiliations:** 1 eritus Professor of Oral Medicine and Pathology. Department of Stomatology. Faculty of Medicine and Nursing. University of the Basque Country/EHU. Leioa. Spain

## Abstract

**Background:**

Oral leukoplakia is the most frequent and representative potentially malignant disorder of what is known as oral precancer. Since the first descriptions, this pathology, which initially seems simple, has been the subject of controversy and discussion, and it still maintains multiple unknowns and enigmas to be solved.

**Material and Methods:**

A narrative and integrative review of the epidemiological, pathogenetic, diagnostic, prognostic and therapeutic aspects of this important oral disorder has been carried out.

**Results:**

Oral leukoplakia still presents multiple enigmas regarding its actual epidemiology, its multifactorial and multistage pathogenesis, its definition and diagnosis, its malignant development and its treatment.

**Conclusions:**

We must conduct well-designed prospective studies on this fascinating oral pathology, on well-diagnosed clinical cases with clinicopathological criteria agreed and accepted by the scientific community. Only in this way will we be able to clarify the enigmas it still presents.

** Key words:**Oral leukoplakia, enigma, pathogenesis, diagnosis, prognosis, treatment.

## Introduction

Oral leukoplakia (OL) is the most frequent and emblematic oral potentially malignant disorder (OPMD). After two centuries being described as such, we still have to consider it ‘enigmatic’, as it still holds many uncertainties regarding very important aspects like: why and how it develops?, why and how it malignizes?, how it can be prevented?, how it should be treated? .....

My first contact with OL was almost 50 years ago, during my pathology residency before becoming a stomatologist, looking under the microscope at ‘oral keratoses’ and ‘oral epidermoid carcinomas’, as we then called them. It was through the preparation of a lecture given by my wife that I learned about the work of Pindborg, Kramer, Silverman, van der Waal, Warnakulasuriya and other great masters. Since then, OL has been a very important part of my work as a healthcare professional, clinician, pathologist, university lecturer and researcher.

OL is undoubtedly the most representative OPMD. To contextualize its importance, we need only refer to its potential consequence, oral squamous cell carcinoma (OSCC), which in the 21st century still shows high morbidity and an unaccepTable mortality. A significant percentage of these carcinomas will develop through OL. Therefore, proper diagnosis and management of OL is essential to improve the prognosis of oral cancer (1).

Oral diseases are as old as humankind and their characteristics have been described for a long time. However, there are no specific references to OL until the 19th century, when Professor Schwimmer, in 1877, made the first publication on: ‘idiopathic mucous plaques of the oral cavity: oral leukoplakia’ (2). From that moment on, the clinicopathological and scientific development of this disorder began, especially since it became the direct responsibility of health professionals involved in oral health. In this scenario, OL was placed as an oral precancerous lesion in what we described as ‘oral precancer’, differentiating ‘lesions and diseases’. Since 2005, these pathologies receive a more appropriate denomination of ‘oral potentially malignant disorders’ (3).

## Material and Methods

A narrative and integrative review of oral leukoplakia has been carried out. We have reviewed its main aspects: terminology and history, etiopathogenic, clinical, histopathological, diagnostic, prognostic and therapeutic.

A comprehensive literature search was carried out in the PubMed database, using the keywords: ‘oral’, ‘leukoplakia’, ‘pathogenesis’, ‘genetics’, ‘clinical’, ‘pathology’, ‘diagnosis’, ‘malignancy’, ‘prognosis’, and ‘treatment’.

The main data collected on this oral pathology were structured and written according to the personal opinion of the author responsible for this article.

## Results

- Epidemiological aspects

To date, we still do not know the real epidemiology of OL, and very heterogeneous results have already been reported (0.33-11.74%), with an overall prevalence of 3.41% (4,5). This variability would be related to population characteristics, socio-cultural factors, toxic habits, dietary and genetic aspects, etc., and also to that inherent to the diagnosis of OL itself, which in many cases is carried out under non-consensual and non-standardized criteria, which still represents a serious handicap in order to clarify the many of the enigmas of OL.

From the first clinical studies on OL, it was found that this disorder was more frequent in men, over 60 years of age, generally tobacco and alcohol consumers. However, we now know that this pathology can also develop in women, younger adults and non-smokers (6,7).

- The etiopathogenesis

We still do not know much about the true etiopathogenesis of OL and whether the mechanisms involved in its development are always the same. In some cases, the cause, or rather, the causes remain unknown and they are still ‘idiopathic’, as professor Schwimmer stated more than two centuries ago. Many times, there are known risk factors such as the consumption of tobacco, alcohol, betel, etc., or others like oral trauma, chronic inflammation, infections, deficiencies, etc. These are involved to a greater or lesser extent in its genesis, which for the moment must be described as multifactorial. Several elements, including those associated with genetic susceptibility, will make it possible for an OL to develop in one person and not in another, apparently similar, individua. Therefore, we can say: ‘oral leukoplakia is not for those who want to have it, but for those who can have it’.

An example of the complex pathogenesis of OL is its association with periodontal disease, which as a chronic inflammatory disorder is capable of inducing epithelial proliferation and releasing cytokines, growth factors, prostaglandins, enzymes, etc. In addition, patients with OL present an oral dysbiosis with oncogenic periodontopathogens such as *Porphyromonas *gingivalis** or *Fusobacterium nucleatum* (8).

The year 1953 was very important for the pathogenic recognition of oral cancer and oral precancer, as the theory of ‘field cancerization’ was published. This proved that oral cancer develops in large multifocal areas, surrounded by abnormal mucosa (9). The helical structure of DNA was also described at the same time, allowing spectacular advances in genetic knowledge (10). Since then, a multitude of genetic, epigenetic and molecular studies on oral carcinogenesis and OL have been and are being carried out. Subsequent research has shown that, what initially seemed simple to explain by progressive accumulation of genetic alterations, associated with a clinicopathological evolution from normal mucosa to OL and ending in OSCC (11), is not always the case. The pathogenesis of OSCC and OL, both malignant and non-malignant, are complex, and although they derive from the squamous mucosa like those in other locations, they are neither unique nor simple, so they should not be mixed or generalized.

Many studies have been performed looking for biomarkers for OL, ‘drivers’ and not just ‘passengers’, and a multitude of genetic alterations with insertions, deletions and mutations have been described, which have shown different levels of evidence. Much research has also been done on the expression of certain oncological proteins such as p53, p16, p14, different cytokeratins, telomerases, cyclins, etc., with partial results and debaTable evidence as prognostic markers (12). An ongoing study of OL has been the presence of deletions in the 3p14 and 9p21 fragments, which are associated with an increased risk of malignancy (13). Copy number variations in certain gene regions, DNA promoter hypermethylation, ploidy analysis and heterozygosity losses have shown promising results to predict malignant development too (14,15). However, the scientific fiasco of some publications that went so far as to recognize a ‘killer aneuploid leukoplakia’ did much harm to OL research and to all of us who are dedicated to studying OPMDs.

The true pathogenesis of OL and its potential malignant development remains a great challenge. Although malignancy may occur by random clonal neutral evolution, we should be able to discover biomarkers capable of predicting malignant progression and to understand the mechanisms by which it occurs. I am confident that the more we delve into the genetic and molecular basis, the closer we will come to understanding the complex process of the genesis of OL and its malignization (16).

-Definition and diagnostic aspects

An important enigma of OL derives from the lack of a clear and universally accepted definition for this disorder. This issue affects its correct diagnosis, a fundamental and transcendental aspect in this oral pathology.

Throughout the last century, and so far this century, successive definitions have been proposed, which unfortunately have been and continue to be based on negative aspects, such as: ‘that does not detach’, ‘that is not another oral white disease’, ‘that is not secondary to other causes except tobacco’, ‘that is not always white’, etc., i.e. ‘leukoplakia is any oral white plaque that is not another disease’. In 1963 a group of experts (17) defined it as: ‘a well-demarcated white elevation of the mucosa of 5 mm or more, which cannot be removed and which cannot be attributed to any other disease’. Subsequently, part of the same group (18) redefined it as: ‘a white patch or plaque that cannot be characterized by clinical or pathological examination as any other disease and whose appearance or origin is not associated with any physical or chemical causation other than tobacco use’. This definition was clearly erroneous, pointing to tobacco as the sole pathogenic factor of OL. In 1996 (19), it was redefined as: ‘predominantly white lesion of the oral mucosa that cannot be characterized as any other defined lesion and some will progress to cancer’, consolidating the clinical subtypes. Following the 2005 London Workshop (3), it was modified as: ‘white plaques of questionable risk having excluded other known diseases or disorders that do not carry an increased risk of cancer’. Oral precancer was also grouped and renamed as ‘oral potentially malignant disorders’. After 15 years, I had the opportunity to participate in the WHO Collaborative group meeting for OPMD in Glasgow (20), where we reviewed these disorders, agreed on clinicopathological diagnostic criteria. The definition of OL was slightly modified as: ‘predominantly white plaque of questionable risk having excluded other known diseases or disorders that do not carry an increased risk of cancer’.

Another aspect regarding the diagnosis of OL needless to be controversial is that this should always be clinicopathological, in order to: differentiating it from other white oral mucosal pathologies, obtaining information on its malignant potential, and treating it appropriately. In OL, it is essential to make a good clinical diagnosis of the lesion(s), based on the clinical history and physical examination. These ought not to be unknown for oral health professionals. To date, there is no evidence to support the use of ancillary methodologies to improve the traditional clinical diagnosis of OL, so it is essential that healthcare professionals are able to recognize the diagnostic signs and symptoms of this pathology (1,20).

OL is a white or predominantly white plaque, easily recognizable and usually asymptomatic, in which it should always be noted whether it is homogeneous or not, as this is a significant prognostic data (20,21). In addition, lesion(s) should be measured and described as single or multifocal, as these are significant prognostic factors (7). Homogeneous leukoplakia is the most common clinical type and appears as a uniform, well-defined white plaque, occasionally with fine fissures. Non-homogeneous leukoplakia holds a higher risk of malignization, and is not uniform, showing indistinct borders, and has three recognized clinical features: erythroleukoplakic, nodular or mottled and verrucous (20).

Nowadays, the appearance of more than two leukoplakia lesions in a patient is a transcendental clinical fact, as the diagnosis will change from ‘conventional oral leukoplakia’ to ‘proliferative (multifocal) verrucous leukoplakia’. In 1985 Hansen, Olson and Silverman (22) first described a multifocal, proliferative, persistent, irreversible, recurrent, therapy-resistant, leukoplakic mucosal disorder, that affects mostly older women and non-smokers. This disorder is most frequently located on the gingiva and have a high risk of malignancy. Since that first description, a lot of information and research has been gathered, which unfortunately has not solved the enigmas that this important and particular OPMD still presents. In 2022, in view of the existing prognostic controversy, we conducted a meta-analytical review and proposed simple diagnostic criteria, with the aim of favoring its prospective diagnosis and monitoring and avoiding under-diagnosis (7).

To reach a final diagnosis of OL, oral disorders that also present as ‘white lesions’ must be discarded candidiasis, alveolar keratosis, white sponge nevus, leukoedema, oral lichenoid disease, lupus erythematosus, hairy leukoplakia, nicotinic palatitis, geographic tongue; etc. Nevertheless, the most important diagnosis that must always be ruled out in this pathology is squamous cell carcinoma, both conventional and verrucous (20).

After reaching a ‘provisional’ clinical diagnosis of OL, all cases should be analyzed histopathologically in order to obtain a ‘definitive’ diagnosis (23). The reasons for always performing a biopsy in OL are: a) to confirm the provisional clinical diagnosis, b) to rule out other oral mucosal disorders, and c) to assess the presence of epithelial dysplasia. Biopsy remains the gold standard technique for reaching a definitive diagnosis in all OPMDs. Furthermore, in some cases, a single incisional biopsy may not be enough and/or may lead to underdiagnoses. Thus, as many biopsies as clinically suspicious areas of OL should be performed (20).

Microscopic histological examination of OL will show non-specific changes of the squamous epithelium and chorionic connective tissue: ortho- and/or para-hyperkeratosis, granulosis, acanthosis, atrophy, crestal growth or flattening, chronic lymphoplasmacytic inflammation, vascular proliferation in the chorion. Sometimes cytological and architectural dysplastic changes are displayed, which the pathologist must recognize, assess and grade (24).

Epithelial dysplasia remains the most important prognostic data when assessing the risk of malignant development of OL (21). Although it is the ‘gold standard prognostic assessment’, it is still a source of controversy and questionable results, as it has significant subjectivity and high inter- and intra-observer variability (24).

‘Oral epithelial dysplasia’ is not a clinical entity, it is a histopathological morphological aspect resulting from a variable combination of microscopic modifications indicative of alterations in epithelial cell maturation and proliferation. Therefore, it does not make sense to consider it or treat it as if it were a clinical disorder. Nowadays inappropriate terms such as ‘lichenoid dysplasia’ or ‘lichenoid proliferative leukoplakia’ have been described, which should not be used in this pathology. Using these terms is a mistake that may lead to clinicopathological underdiagnoses and incorrect therapeutic or control measures (25).

In an attempt to improve the classical classification of epithelial dysplasia as mild, moderate and severe, and in order to help clinicians make therapeutic decisions, a new binary classification into low-grade and high-grade dysplasia was proposed in 2006 (26). However, this simplifying proposal, which was initially well received, is now being questioned, as it does not improve the classical system. A combination of both systems would be better (27). In 2022, a new grading system was introduced with specific data: bulbous ridges, hyperchromatism, loss of cohesion and stratification, suprabasal mitoses, and nuclear pleomorphism. This new proposal has shown a good predictive relationship for malignancy and recurrence (28). It has also been suggested to analyze the presence of ‘differentiated dysplasia’, based on differentiated vulvar neoplasia, associated with an increased risk of malignancy (29). More recently, an artificial intelligence process has been developed, which, using morphological and spatial features and emulating histological markers, could predict malignant progression of OL based on the detected epithelial dysplastic alterations. This automated predictive algorithm would show performance comparable to that of the pathologist (30).

- Malignant development

We have known for almost two centuries that OL is a potentially malignant disorder, and since then a multitude of studies have been conducted in an attempt to determine the true magnitude of this risk and the factors linked to malignancy (31). The percentage of malignant development obtained in the classic studies has widely varied (0.09-38.5%). This is a clear sign of its heterogeneity, leading to new analyses with more precise and reliable methodologies, trying to determine the real risk of this malignant development, which is nowadays considered to be between 5 and 10% (20,32).

In 2021, we conducted a review and meta-analysis of the studies published between 2015 and 2020, in which we found a malignant development rate of 9.8% (21). Significant risk factors were: advanced age (over 50 years), female gender, tongue location, non-homogeneous clinical type, and presence of epithelial dysplasia. Interestingly, other factors such as large size or tobacco use were not significant. In September 2024, a rigorous meta-analytical study (32) was published with the aim of updating the evidence on malignant transformation of OL, including all studies published since 1934. In this study, the percentage of malignant transformation was 6.64%, with no significant variations depending on the period of the studies analyzed. The risk factors that were significant in this study were: non-homogeneous clinical type, large size, lingual border location, tobacco consumption and epithelial dysplasia.

These analyses, methodologically appropriate, highlight the great variability that still exists in what is the most important aspect of OL. I believe that this is mainly due to the fact that the studies analyzed have been carried out on cases diagnosed with non-homogeneous criteria, with varying control periods, using different treatments, etc. It is therefore very important that we carry out well-designed clinicopathological studies on well-diagnosed and controlled cases of OL, following agreed criteria.

Therapeutic aspects

Patients diagnosed with OL should be informed by the healthcare professional about the pathology they are suffering and about its clinical and biological aspects. Even though malignant development may only occur in some cases, they should always be aware of the risk of malignization, as this is the key element for making decisions about treatment and control.

Unfortunately, there are still no consensus guidelines for the treatment of OL (33,34), with very limited scientific evidence available on the multiple medical and surgical therapies tested. No treatment has been effective in preventing the malignant development of OL, and although some therapies appear to resolve lesions, recurrences and adverse effects are common (35). Multiple systemic and topical agents have been evaluated, including: vitamin A, retinoids, carotenoids, bleomycin, protease inhibitors, herbal extracts and mixtures, and many others, although none have provided significant evidence of a reduction in the risk of malignant development over placebo (35).

There is also no evidence that cessation of recognized risk factors, such as tobacco use, leads to resolution of lesions, although some OLs will disappear after smoking cessation (36).

The therapeutic approach that has prevailed in OL so far is to treat all leukoplakia with high-grade dysplasia surgically, although there is insufficient scientific evidence to support this (35). Conventional surgical excision with margins remains the first-line treatment for ‘high-risk’ leukoplakias, as it allows removal of the entire lesion and correct histopathological assessment. However, we know that any OL can become malignant, including ‘low-risk’ ones, and that the risk of recurrence and malignant development does not disappear even when the leukoplakia has been completely removed (37). Recently, initial results of a randomized clinical trial on the surgical treatment of OL have been published (38), indicating a higher risk of malignancy for OL with epithelial dysplasia in the ‘wait-and-see’ group than for OL with epithelial dysplasia in the ‘surgical removal’ group.

Another very important aspect of this pathology that has not yet been fully resolved concerns the monitoring that should be carried out in these patients after the diagnosis of OL. Based on current knowledge, I believe all patients diagnosed with OL, whether treated or not, should be monitored periodically for life, at intervals that should vary depending on the diagnosis, the risk factors present and the evolution of each case. Given that we still do not have enough evidence on any treatment capable of preventing its malignant development, I must again point out the need for quality research to adequately evaluate the efficacy and safety of new therapies (32,39).

Finally, as a conclusion of this personal review on the ancient but still ‘enigmatic oral leukoplakia’, I would like to emphasize the need to conduct well-designed prospective studies on this important and fascinating oral pathology, performed on well-diagnosed clinical cases with clinicopathological criteria agreed and accepted by the scientific community. Only this will allow us to recognize significant data and markers involved on its genesis, malignant development and appropriate therapy.
